# Multiples of multiple: Case series of mirror aneurysms and review of literature

**DOI:** 10.1016/j.ijscr.2019.07.037

**Published:** 2019-07-22

**Authors:** Niranjana Rajagopal, Yasuhiro Yamada, Arun Balaji, Tsukasa Kawase, Yoko Kato

**Affiliations:** a Department of Neurosurgery, Sri Sathya Sai Institute of Higher Medical Sciences, Bangalore, India; b Department of Neurosurgery, Fujita Health University, Banbuntane Hotokukai Hospital, Nagoya, Japan; c Department of Neurosurgery, Kovai Medical Center and Hospital, Coimbatore, Tamil Nadu, India

**Keywords:** Multiple intracranial mirror aneurysms, Embryology, Treatment options

## Abstract

**Introduction:**

Mirror aneurysms are a rare subtype of multiple aneurysms, located in identical or adjacent arterial segment bilaterally. We report a case series of 3 such patients amongst whom one of them had 3 sets of mirror aneurysms and the other patient had 2 sets of mirror aneurysm on the same arterial segment which has not been reported till date.

**Methods:**

A retrospective analysis of 3 patients with incidentally detected multiple mirror aneurysms, who were treated with microsurgical clipping and coiling, was conducted. A systematic search was performed using the PUBMED database and relevant articles were reviewed with particular attention to incidence, associated conditions, risk factors and management strategies. Written informed consent was obtained from all of the patients for publication of this case report and accompanying images. A copy of the written consent is available for review by the Editor-in-Chief of this journal on request. This research work has been reported in line with the PROCESS criteria (Agha et al., 2018).

**Results:**

All the 3 patients were females with incidentally detected multiple mirror aneurysms. All the 3 patients were known Hypertensives. All the aneurysms were successfully clipped by a multistage, bilateral craniotomy except the one in the cavernous ICA, for which an endovascular procedure is planned. All of them had an uneventful postoperative course with the CT angiogram showing obliteration of all the clipped aneurysms except the one in the cavernous ICA.

**Conclusions:**

Multiple mirror aneurysms represent a rare occurrence of a diverse pathology. Both these described types of cases have not been reported so far in the literature. The treatment strategy for mirror aneurysms should be determined individually based on the location, size, and morphology of the aneurysms, as well as, on the clinical manifestations of each patient.

## Highlights

• Incidentally detected unruptured mirror intracranial aneurysms are on the rise due to advanced imaging modalities.
• Further studies are required to elucidate theories of development, inheritance pattern and the need for screening of 1st-degree relatives with twin aneurysms.
• Treatment strategy for mirror aneurysms should be customised based on the location, size, morphology and clinical manifestations of each patient.
• Randomised control trials that compare [single/multistage] clipping and coiling of mirror aneurysms are necessary to decide the best intervention strategy.

## 1 Introduction

Mirror or twin aneurysms are a rare subtype of multiple aneurysms, located in identical or adjacent arterial segment bilaterally. Their incidence varies from 5% to 40% of all patients with multiple aneurysms. The commonest anatomical location is the middle cerebral artery (MCA), followed by the Internal cerebral artery (ICA) and the Posterior communicating artery (Pcom). They are present in the other vessels as well but are more uncommon [[1], [2], [3], [4], [5]]. Multiple sets of mirror aneurysms in the same patient are exceedingly rare. There are a total of 6 such case reports available in the literature to date [1,4,[6], [7], [8]]. We report 3 such patients amongst whom, one of them had 3 sets of mirror aneurysms and the other patient had 2 sets of mirror aneurysm on the same arterial segment which has not been reported till date.

## 2 Methods

A retrospective analysis of 3 patients with incidentally detected multiple mirror aneurysms, who were treated with microsurgical clipping and coiling, was conducted. A systematic search was performed using the PUBMED database. Relevant articles were reviewed with particular attention to incidence, associated conditions, risk factors and management strategies. Written informed consent was obtained from all of the patients for publication of this case report and accompanying images. A copy of the written consent is available for review by the Editor-in-Chief of this journal on request. This research work has been reported in line with the PROCESS criteria [28].

## 3 Illustrative case 1

**Fig. 1:**
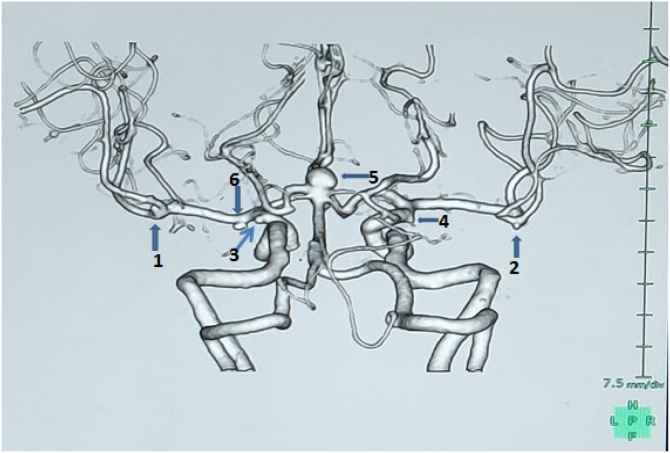
Case 1 aneurysms with their sizes:- **1,2** – bilateral MCA bifurcation aneurysms(right- 2 mm, left- 0.4 mm); **3,4** – bilateral Anterior choroidal artery aneurysms(right-3 mm, left- 2 mm); **5** - basilar top (6.4 mm); **6** - left M1 aneurysm(3 mm).

**Fig. 2:**
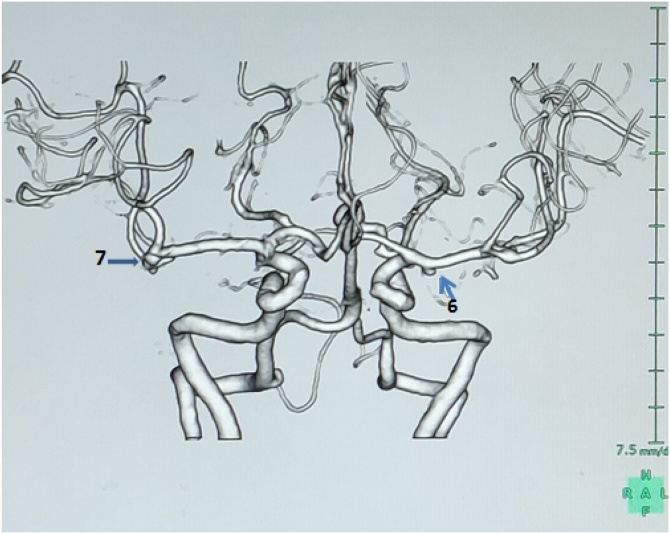
Case 1 aneurysms with their sizes:- **6** - left M1 aneurysm(3 mm); **7** – Right M1 aneurysm(3 mm).

A 57 years old lady, who was a known case of hypertension on medications, was incidentally detected to have multiple aneurysms when she was screened for the same as she had a family history of subarachnoid haemorrhage but not of any detected aneurysms. She had no history of smoking, alcohol abuse or history of any syndromes. On examination, she did not have any neurological deficits. She underwent CT angiogram of the brain with three-dimensional reconstructions and a 4-vessel DSA which demonstrated the following aneurysms: - basilar top, bilateral M1, bilateral MCA bifurcation and bilateral anterior choroidal artery aneurysms (Fig. 1, Fig. 2). There were a total of 7 aneurysms in the patient. After careful evaluation of the radiologic images, we decided to attempt a 2 stage surgical procedure and clipping of all the aneurysms. She initially underwent a left pterional craniotomy and clipping of the basilar top, left anterior choroidal, M1 and MCA bifurcation aneurysms. She underwent the second surgery after 1 month which was a right pterional and trans-sylvian approach similar to the first one and clipping of right anterior choroidal, M1 and MCA bifurcation aneurysms. The post-operative courses after both the procedures were uneventful. The post-operative CT angiogram showed complete exclusion of all the aneurysms while preserving the normal flow.

## 4 Illustrative case 2

**Fig. 3:**
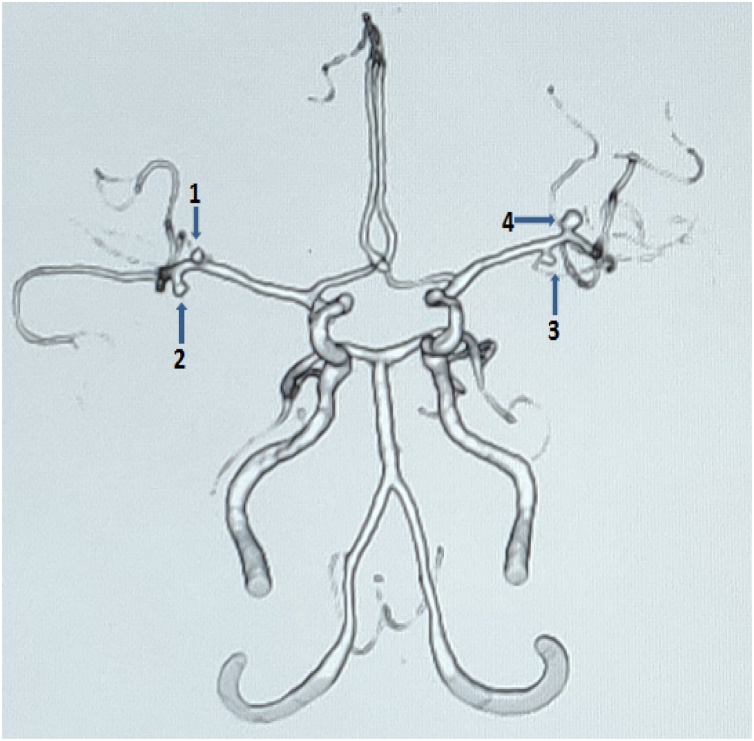
Case 2 aneurysms with their sizes:-Bilateral MCA aneurysms (**1**-2.3 mm, **2**- 2.6 mm, **3**- 2.4 mm, **4** – 3.2 mm).

**Fig. 4:**
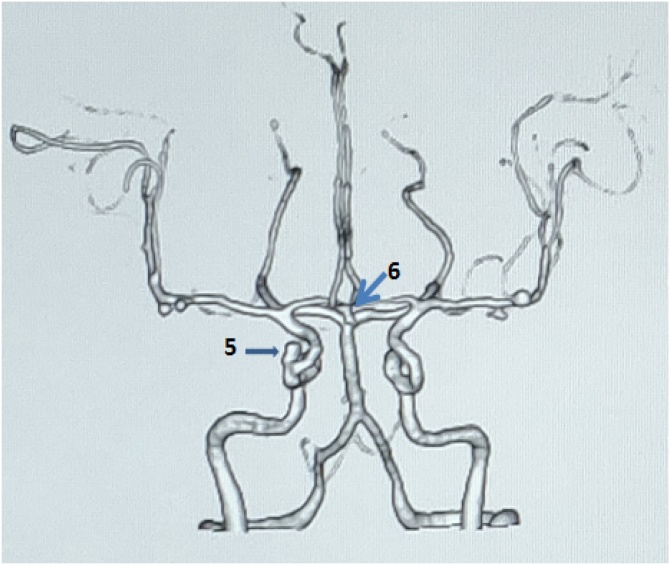
Case 2 aneurysms with their sizes:- **6** – Acom aneurysm (3 mm); **7** – Right Cavernous ICA aneurysm (3.2 mm).

A 63 years old lady, who was a known case of hypertension on medications, was incidentally detected to have multiple aneurysms when she was evaluated for a nonspecific type of headache. She had no family history of SAH or aneurysms. She had no past history of smoking, alcohol abuse or any syndromes. On examination, she did not have any neurological deficits. She underwent CT angiogram of the brain with three- dimensional reconstructions and a 4-vessel DSA which demonstrated the following aneurysms:- Acom, 2 sets of bilateral M1 and a right cavernous ICA aneurysms (Fig. 3, Fig. 4). There were a total of 6 aneurysms in the patient. We decided to attempt a 2 stage surgical procedure and clipping similar to the previous case. She initially underwent a right pterional craniotomy and clipping of both the MCA and Acom artery aneurysms. The right cavernous ICA aneurysm was wrapped. She underwent the second surgery after 1 month which was a left pterional and clipping of the left MCA aneurysms. The post-operative courses after both the procedures were uneventful. The post operative CT angiogram showed complete exclusion of all the aneurysms except the right cavernous ICA aneurysm while preserving the normal flow. She is planned for an endovascular procedure for the cavernous ICA aneurysm.

## 5 Illustrative case 3

A 74 years old lady, who was a known case of hypertension on medications, was incidentally detected to have multiple aneurysms when she was screened for the same as she had a family history of subarachnoid haemorrhage but not of any detected aneurysms. She had no history of smoking, alcohol abuse or history of any syndromes. On examination she did not have any neurological deficits. She underwent CT angiogram of the brain with three-dimensional reconstructions and a 4-vessel DSA which demonstrated the following aneurysms: - bilateral M1, bilateral anterior choroidal artery and left ICA bifurcation aneurysms. There were a total of 5 aneurysms in this patient. After careful evaluation of the radiologic images, we decided to attempt a 2 stage surgical procedure and clipping of all the aneurysms. She initially underwent a left pterional craniotomy and clipping of the left M1, ICA bifurcation and choroidal artery aneurysms followed by a similar procedure on the right after 1 month. The post-operative course after both the procedures was uneventful. The post-operative CT angiogram showed complete exclusion of all the aneurysms while preserving the normal flow.

**Fig. 5:**
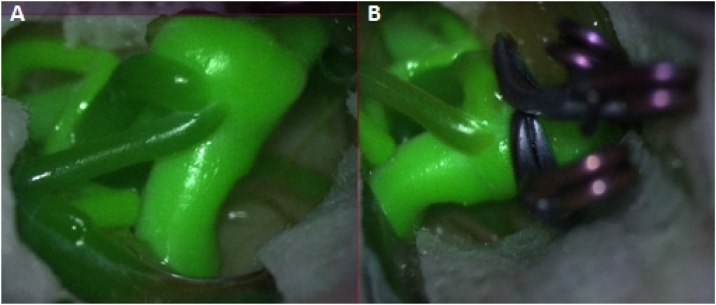
A) Pre clipping DIVA showing the aneurysm and the adjacent vessels. B) Post clipping DIVA shows no residual aneurysms with intact adjacent vessels.

All the surgeries were performed by the senior most professor in the hospital. Intraoperatively, we used electrophysiological monitoring like MEP, indocyanine green videoangiography with DIVA (Fig. 5) for an intraoperative assessment of the occlusion of the aneurysms and the preservation of the blood flow in parent/ branching vessels and doppler in all the cases. There was no intraoperative rupture in any of the procedures.

## 6 Discussion

Multiple aneurysms are a heterogenous group of patients within the broader group of intracranial aneurysms accounting for 12.9%–26.4% of all aneurysms [9]. Mirror aneurysms is a small subset amongst multiple aneurysms which represent less than 5% of all intracranial aneurysms of which multiple mirror aneurysms are even rarer [4,5]. They are being increasingly detected in recent times due to the wider availability and advancements in imaging modalities [3,8]. Women have a higher incidence of aneurysms than men in general, especially older women (post-menopause), more so in mirror aneurysms. When compared with premenopausal patients, a greater percentage of post-menopausal ladies have multiple aneurysms. It has been postulated that lack of Oestrogen could be a causative factor for this, but there are no studies till date to prove this [3,4,[9], [10], [11]]. Smoking is another important causative factor for the formation of multiple or mirror aneurysms. Along with the pathologic changes induced by smoking, it is also known to have anti-oestrogen features, thus promoting the formation of an aneurysm in menopausal women [3]. Thus a thorough evaluation to exclude mirror aneurysms must be done in patients presenting with aneurysms in MCA, ICA or Pcom arteries, especially in menopausal women and smokers.‬ ‬‬‬‬‬

Various authors have postulated an embryological basis for mirror aneurysms. Baccin et al. [1] has discussed in detail about the development of intracranial basal arteries from the 3 embryological segments – Procencephalic, mesencephalic and rhombencephalic. The intracranial vascular system is composed of different segments embryologically. This concept of “segmental identity’’ suggests the vulnerability of different regions of the intracranial vascular tree to specific triggers. Therefore, certain diseases may involve specific regions of the vascular system and spare others. The very fact that this pathology evolves in identical or adjacent arterial segments bilaterally suggests that the insult was specific during the cephalic segmentation phase. If they are present in non-adjacent segments, they probably occur due to the insult occurring prior to the cephalic segmentation. It also suggests a familial or a genetic association [1,4,8], though neither of our patients had a family history or history of any genetic syndromes. Among the six cases of multiple mirror aneurysms found in the literature (Table 1), the aneurysms were located in identical segments in four patients and nonadjacent segments in the other two. In two of our patients, the aneurysms were mirror images of their counterparts, whereas, in the third case which had 3 sets of mirror aneurysms, one set amongst them was not categorically mirror but in the same arterial segment though. In all the 3 of our cases, the size, shape and direction of aneurysms were mirror images of their counterparts. Another rarity in our series was that Case 2 had multiple sets of mirror aneurysms in the same arterial segment which has not been reported till date.

**Table 1:** Review of literature on multiple mirror aneurysms.

Author / Year	Age / Sex	Presentation	Location of mirror aneurysms	Treatment planned	Procedure	Other aneurysms	Embryology	Outcome
Yamada et al. 2000	54/ Male	First presentation : Left cavernous syndrome Second presentation : SAH	Cavernous ICA and vertebral artery	Multistaged	Permanent ligation of left carotid artery	None	Non adjacent segments [prosencephalic and rhombencephalic]	Recovered after ligation but died after the SAH from vertebral artery
Baccin et al. 2006	55/ Female	SAH	MCA bifurcation and PICA	Multistaged	Left MCA clipping	ACom	Non adjacent segments [prosencephalic and rhombencephalic]	Partial recovery
Xu et al. 2013	71/ Female	SAH	PCom and MCA bifurcation	Single stage [bilateral craniotomy]	Clipping of all aneurysms	Right M2 and left M1 aneurysms	Identical segments [Procencephalic]	Partial recovery in immediate post operative period. Doing well at the end of 1 year
Maiti et al. 2015	73/ Female	Right MCA stroke	Cavernous ICA and MCA bifurcation	Multistaged	Clipping of Right MCA aneurysm	Right M1, M3 and left A1 aneurysms	Identical segments [Procencephalic]	Partial recovery of hemiparesis. Refused second surgery.

Wang et al. 2017	50/ Female	SAH	Pcom and peri callossal/ callosomarginal	Multistaged	Clipping and coiling	None	Identical segments [Procencephalic]	Partial recovery
	38/ Male	Asymptomatic	MCA and peri callossal/ callosomarginal	Multistaged	Clipping and coiling	none	Identical segments [Procencephalic]	No deficits postoperatively

Present series	57/ Female	Asymptomatic	M1, MCA bifurcation and Anterior choroidal	Multistaged	Clipping of all aneurysms	Basilar Top	Non adjacent segments [prosencephalic and mesencephalic]	No deficits postoperatively
	63/ Female	Asymptomatic	2 sets of M1 aneurysms	Multistaged	Clipping and coiling	ACom, Right cavernous ICA	Identical segments [Procencephalic]	No deficits postoperatively
	74/ Male	Asymptomatic	M1 and Anterior choroidal	Multistaged	Clipping of all aneurysms	Left ICA bifurcation	Identical segments [Procencephalic]	No deficits postoperatively

The mere presence of mirror aneurysms is not a predictor of poor outcome or SAH [8,12]. The management principles of mirror aneurysms are not very different from that of multiple aneurysms. Mirror aneurysms can be managed by conservative approach, surgery, endovascular interventions or a combination of these depending on various factors such as age, location, size, morphology, clinical presentation, medical co-morbidities, available expertise, cost and infrastructure [8,[13], [14], [15], [16], [17], [18]]. The management requires a balance between the two ideologies namely: a single stage of treatment that eliminates misidentification and treats all the lesions and a multi-staged treatment that avoids the extensive cumulative manipulation and retraction injury. Thus treatment has to be individualized with careful planning as to which aneurysm should be addressed first, subsequent sequence and the appropriate position to better visualize the neck of aneurysms to reduce exposure. Aneurysms that have bled have to be treated as a priority. For this, lateralisation of bleed in a mirror aneurysms is imperative, which is sometimes tricky even with the latest diagnostic imaging modalities [9,16,17,[19], [20], [21], [22], [23]]. Zderkiewicz et al. [24] found that the accuracy of the examination method (neurologic examination, DSA, CT scan, and/or intraoperative evaluation), in terms of revealing the responsible aneurysms, was only 72.16%, hence a high index of suspicion and skill is required to diagnose these lesions. Bilateral unruptured aneurysms can be treated surgically with a single-stage or a multiple staged approach depending on the clinical presentation, type of intervention, age, patient preference, cost, facilities available [17,25]. Single-stage approach may be further divided into unilateral and bilateral craniotomies depending on the location of the contralateral aneurysms, its size and shape [16,17,[19], [20], [21], [22], [23]].

Despite recent advances in knowledge and techniques, treatment strategies for mirror aneurysms remain controversial. Both single and multi staged treatments are associated with good results. Hence, treatment has to be customised to every case accordingly [18,26,27].

## 7 Conclusion

Multiple mirror aneurysms represent a rare occurrence of a diverse pathology. We have described 3 such cases of which one had 3 sets of mirror aneurysms and another case had 2 sets in the same arterial segment. Both these types have not been reported so far in the literature. Further studies are required to elucidate the possible theories of development, inheritance pattern, and association of these lesions and the need for screening of 1st- degree relatives with twin aneurysms. The treatment strategy for mirror aneurysms should be determined individually based on the location, size, and morphology of the aneurysms, as well as, on the clinical manifestations of each patient.

## Funding

Nil.

## Ethical approval

The paper was on a retrospective case series. The procedure followed was the standard treatment of care as highlighted in the manuscript. There was no testing of any form done. Hence there was no nee of an ethics approval.

## Consent

Written informed consent was obtained from all of the patients for publication of this case report and accompanying images. A copy of the written consent is available for review by the Editor-in-Chief of this journal on request.

## Author contribution

Dr. Niranjana - data collection, data analysis, writing of the paper.

Dr. Arun Balaji - data collection and inputs.

Dr. Yamada - guidance for writing.

Dr. Kawase- Guidance and editing.

DR kato - study concept.

## Registration of research studies

IJS Publishing Group Ltd.

Invoice #09C57F19-0001

Receipt #2944-4204.

Research Registry

UIN : researchregistry4800.

## Guarantor

I take complete responsibility for the work and conduct of the study - Dr Niranjana Rajagopal.

## Provenance and peer review

Not commissioned.

## Declaration of Competing Interest

There are no conflicts of interest.
